# Fucoxanthin-Loaded Solid Lipid Nanoparticles Exert Potent Therapeutic Efficacy in Combating High-Fat Diet Induced Obesity in Mice

**DOI:** 10.3390/ijms26115249

**Published:** 2025-05-29

**Authors:** Lijun Ding, Xiao Luo, Weijia Wen

**Affiliations:** 1 Thrust of Bioscience and Biomedical Engineering, The Hong Kong University of Science and Technology (Guangzhou), Nansha, Guangzhou 511400, China; lding472@connect.hkust-gz.edu.cn; 2 Department of Physics, The Hong Kong University of Science and Technology, Hong Kong 999077, China; xluoay@connect.ust.hk; 3 Thrust of Advanced Materials, The Hong Kong University of Science and Technology (Guangzhou), Guangzhou 511400, China

**Keywords:** fucoxanthin, solid lipid nanoparticle (SLN), high-fat diet, efficacy, anti-obesity

## Abstract

Obesity and associated metabolic disorders pose significant health challenges. Fucoxanthin, a lipophilic compound, has shown promising anti-obesity potential, but its poor solubility and bioavailability limit therapeutic efficacy. The successful formulation of solid lipid nanoparticles (SLNs) amplified fucoxanthin’s efficacy in mitigating obesity and the associated metabolic dysregulation. High-fat diet (HFD)-induced obese mice were treated with free fucoxanthin, lyophilized SLNs (L-SLN), and dispersed SLNs (D-SLN) loaded with fucoxanthin. The intervention with D-SLN demonstrated the most significant reduction in body weight gain (29.94%) and fat mass gain (61.80%) compared to the HFD group (*p* < 0.05), alongside notable improvements in metabolic indicators including fasting blood glucose, liver enzymes, lipid profile, and inflammatory markers such as leptin and monocyte chemoattractant protein 1 (MCP-1) levels. Histopathological evaluation corroborated these findings, showing highly reduced hepatic lipid droplet accumulation and improved adipocyte and testicular morphology. This advancement paved the way for translating fucoxanthin into a clinically effective anti-obesity agent.

## 1. Introduction

Obesity has emerged as a critical global health concern, reaching epidemic proportions in recent decades. Data from the World Health Organization (WHO) indicate that 2.5 billion adults were classified as overweight in 2022, of whom 890 million were living with obesity [1]. The World Obesity Atlas (2024) projects that by 2035, over 50% of global adults will be overweight or obese [2]. Obesity is not just a cosmetic concern; conditions such as type 2 diabetes, cardiovascular diseases, hypertension, and certain forms of cancer have all been associated with obesity [3]. Moreover, the economic burden of obesity on healthcare systems is substantial. Costs related to treatment, prevention, and loss of productivity are soaring, hence making it a pressing issue that demands urgent attention [4].

**Figure 1:**
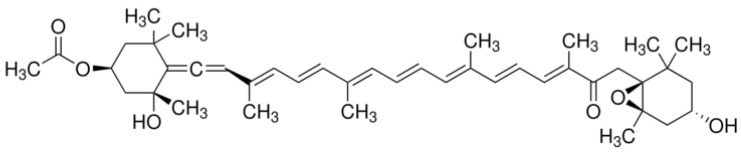
The chemical structure of fucoxanthin.

Given the gravity of these challenges, the search for effective obesity management strategies has become a top priority. In this pursuit, natural bioactive compounds have emerged as a promising area of research. Among them, fucoxanthin, a unique carotenoid predominantly found in brown seaweeds such as *Undaria pinnatifida*, *Laminaria japonica*, and *Sargassum* species, has shown promising anti-obesity and anti-inflammatory properties [5]. Fucoxanthin has a distinct chemical structure characterized by an allenic bond and an epoxide group (Figure 1), which contribute to its diverse biological activities [6].

Numerous in vitro and vivo studies have demonstrated the potential of fucoxanthin in combating obesity [7,8]. In vitro, fucoxanthin has been shown to inhibit the differentiation of pre-adipocytes into mature adipocytes. It acts by modulating key transcription factors involved in adipogenesis, such as peroxisome proliferator-activated receptor gamma (PPARγ), CCAAT/enhancer-binding proteins (C/EBPs), and sterol regulatory element-binding protein 1c (SREBP-1c) [9,10]. In animal models, fucoxanthin supplementation has been reported to reduce body weight gain, decrease adipose tissue mass, and improve lipid metabolism; for example, it induces the expression of uncoupling protein 1 (UCP1) in white adipose tissue, promoting increased energy expenditure and reduced fat accumulation [11]. Additionally, it influences gut microbiota, a critical factor in energy homeostasis and obesity development [12,13].

However, the clinical application of fucoxanthin is significantly hindered by its poor aqueous solubility and low bioavailability, primarily attributed to the rapid degradation of free fucoxanthin in the gastrointestinal tract and suboptimal tissue distribution, which restrict its ability to exert target-specific effects in metabolic tissues such as the liver, adipose, and reproductive organs [14,15]. These limitations result in ineffective outcomes with low-dose supplementation. Studies have shown that dietary concentrations of 0.015–0.03% *w*/*w* failed to reduce adiposity and, in some cases, paradoxically increased body weight by 12.4% compared to controls [16]. Park et al. observed no significant weight loss in mice receiving 0.02% *w*/*w* free fucoxanthin [17], while Maeda et al. reported no changes in body weight or energy intake with 0.1% *w*/*w* fucoxanthin alone or combined with MCT [18].

In an attempt to surmount the barriers impending the efficacy of Fucoxanthin, researchers have explored various strategies. For example, Li et al. [19] reported that studies on fucoxanthin-loaded microcapsules (Fx-MICs) have demonstrated their potential in facilitating weight reduction. In their research, when high-fat diet (HFD)-induced mice were administered Fx-MICs at a dosage of 170 mg/kg/day, a 15.25% reduction in liver weight was observed compared to the HFD-fed control group. Despite the fact that fucoxanthin has demonstrated no observable toxicity in rodent studies, even at a substantially high dose of 2000 mg/kg/BW/day [20,21], factors such as cost-effectiveness and potential safety concerns restrict their long-term practicality at high dosage levels.

Building on the foundation of prior research, our research group previously introduced fucoxanthin-loaded solid lipid nanoparticles (SLNs) [22]. This innovative delivery system represented a significant advancement in in vivo pharmacokinetic studies; the bioavailability of fucoxanthin encapsulated in SLNs was found to be 27-fold higher than that of free fucoxanthin. The current study aims to validate fucoxanthin-SLNs as a novel delivery system that enhances fucoxanthin’s efficacy in mitigating HFD-induced obesity. Our central hypothesis is that SLN encapsulation could overcome gastrointestinal degradation and tissue distribution limitations of free fucoxanthin, thereby amplifying its effects on obesity-related pathophysiology, including hepatic steatosis, adipose tissue inflammation, and reproductive dysfunction. Furthermore, we aim to establish a dosing strategy for fucoxanthin-SLNs. Specifically, we seek to identify a dosage that is significantly lower than that of free fucoxanthin while maintaining or even enhancing the therapeutic efficacy. This approach not only aims to optimize the treatment of obesity and its associated complications but also to mitigate potential risks and costs associated with high-dose therapies.

## 2. Results

### 2.1. Characterization of SLN Formulation

#### 2.1.1. Particle Size, PDI, and Zeta Potential

The lyophilized fucoxanthin-loaded solid lipid nanoparticles (L-SLNs) were reconstituted in ultrapure water, yielding particles with marginally larger diameters compared to freshly dispersed fucoxanthin-loaded solid lipid nanoparticles (D-SLNs). The results from dynamic light scattering (DLS) revealed mean particle sizes of 261.3 ± 3.14 nm for D-SLN and 237.21 ± 1.75 nm for L-SLN (Table 1). Notably, both forms maintained particle sizes with the nanoscale range (<300 nm), a threshold for lymphatic absorption and biological barrier penetration [23], which is critical for stability and bioavailability. Both SLNs maintained a narrow polydispersity index (PDI < 0.25), indicating homogeneous redispersion. Zeta potential values for both SLNs ranged between −30.6 and −32.7 mV (Table 1). These values exceeded the threshold for colloidal stability (±30 mV) [24], confirming colloidal stability due to electrostatic repulsion. Notably, L-SLN retained comparable surface charge to D-SLN, suggesting that the lyophilization process did not significantly alter the surfactant coating or lipid integrity [25].

#### 2.1.2. Encapsulation Efficiency (EE%)

Encapsulation efficiency remained consistently high (>95%) for both SLN forms (Table 1). D-SLN achieved an encapsulation efficiency of 98.17 ± 1.21%, which was slightly higher than L-SLN with an EE of 96.91 ± 2.06%. This 1.26% absolute difference in encapsulation efficiency indicated a marginally better performance of D-SLN in encapsulating fucoxanthin under the same experimental conditions, which was due to minor drug leakage during freeze-drying induced phase transitions [26].

### 2.2. Body Weight Modulation

#### 2.2.1. Dose Form-Dependent Efficacy

**Figure 2:**
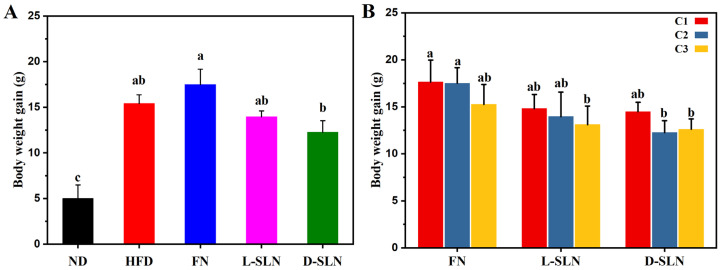
Fucoxanthin formulations attenuate high-fat diet induced weight gain in mice. (**A**) Body weight gain across treatment groups: Normal diet (ND), high-fat diet (HFD), fucoxanthin powder (FN), lyophilized SLN (L-SLN), and fresh SLN dispersion (D-SLN) after 8 weeks of oral administration with middle concentration (66.67 mg/kg). (**B**) Dose-dependent anti-obesity efficacy of FX, L-SLN, and D-SLN (C1: 33.33 mg/kg, C2: 66.67 mg/kg, C3: 100 mg/kg). Data are expressed as mean ± SD, n = 5; bars without sharing the same letter are significantly different (*p* < 0.05).

Following eight weeks of oral administration, significant intergroup variations in body weight gain were observed among mice treated with free fucoxanthin (FN) and fucoxanthin-loaded solid lipid nanoparticles (FN-SLNs). Exemplified by the fucoxanthin intervention groups at middle-concentration of 66.67 mg/kg (Figure 2A), mice on a normal diet (ND) exhibited an average weight gain of 5.00 ± 1.49 g, whereas the high-fat diet (HFD) control group showed a substantial increase of 15.42 ± 0.95 g. The FN intervention resulted in a weight gain of 17.50 ± 1.67 g, slightly higher than the HFD control, while lipid-based solid lipid nanoparticle formulations (L-SLN and D-SLN) demonstrated weight-moderating effects, with gains of 13.96 ± 0.64 g and 12.26 ± 1.26 g, respectively. Quantitative analysis relating to the HFD control revealed significant reductions in weight gain; L-SLN and D-SLN decreased weight gain by 9.47% and 20.49%, respectively. When compared with the FN group, L-SLN and D-SLN induced 20.23% and 29.94% (*p* < 0.05) reductions in weight gain, underscoring the enhanced therapeutic efficacy of nanoparticle encapsulation.

#### 2.2.2. Concentration-Dependent Effects

The experimental outcomes demonstrated a general dose–response relationship, where an increase in treatment concentrations was consistently associated with a decrease in body weight gain across various treatment groups, as illustrated in Figure 2B. The FN group exhibited therapeutic efficacy only at the highest tested concentration, with a mean weight gain of 15.26 ± 2.10 g, which was lower than that of the high-fat diet (HFD) group for 15.42 ± 0.95 g. Specifically, the L-SLN formulation demonstrated a progressive suppression of weight gain, with values decreasing from 14.8 ± 1.50 g to 13.1 ± 1.99 g as the concentration increased. Similarly, the D-SLN formulation showed comparable efficacy but achieved a more substantial decrease in body weight gain. Notably, the concentration-dependent weight suppression effect of the D-SLN formulation reached a plateau at the highest doses. This observation indicated the potential existence of an optimal therapeutic concentration range for the SLN delivery system.

### 2.3. Fat Deposition Analysis

#### 2.3.1. Adipose Tissue Visualization

**Figure 3:**
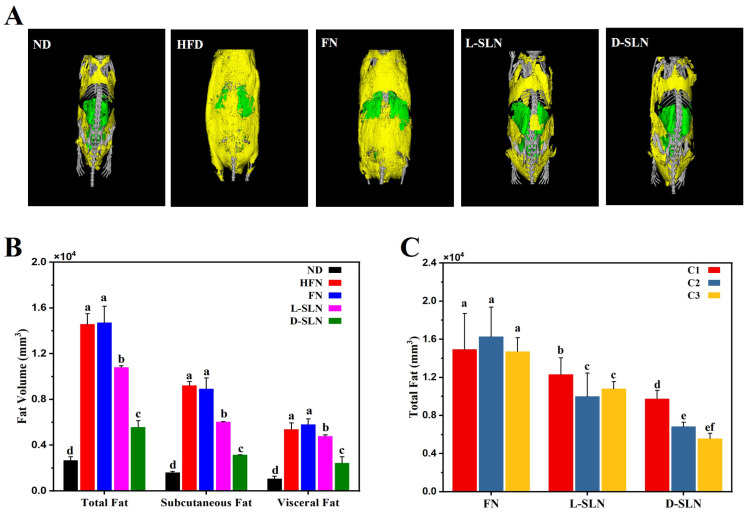
Fat deposition analysis in different intervention groups (ND, HFD, FN, L-SLN, and D-SLN) (**A**) Micro-CT images of adipose tissue, with the yellow color representing subcutaneous fat and the green color representing visceral fat, after 8 weeks of oral administration with middle concentration (66.67 mg/kg). (**B**) Dose-dependent quantification of fat volume (mm^3^), illustrated with the dose concentration of 66.67 mg/kg. (**C**) Concentration-dependent efficacy. Data are expressed as mean ± SD, n = 5; bars without sharing the same letter are significantly different (*p* < 0.05).

The three fucoxanthin dose forms exhibited markedly distinct effects on fat reduction through Micro-CT imaging, exemplified by the fucoxanthin intervention groups at a middle concentration of 66.67 mg/kg (Figure 3A). The normal diet (ND) group exhibited minimal fat deposition, with adipose tissue primarily localized to physiological depots. In contrast, high-fat diet (HFD) mice displayed extensive fat accumulation, particularly in subcutaneous and visceral regions, consistent with severe obesity. Notably, free fucoxanthin (FN) intervention failed to mitigate HFD-induced adiposity, with the fat distribution patterns nearly indistinguishable from the HFD control group. In stark contrast, both SLN formulations (L-SLN and D-SLN) significantly reduced fat deposition. D-SLN demonstrated superior efficacy across all three different dose concentrations. These results underscore that solid lipid nanoparticle engineering was pivotal to overcoming the bioavailability limitations of fucoxanthin.

#### 2.3.2. Formulation and Dose-Dependent Suppression of Adiposity

When comparing the different forms of fucoxanthin delivery systems, for instance, at a dose concentration of 66.67 mg/kg (Figure 3B), the total fat volume in the FN intervention group was 16,262.6 ± 3106.0 mm^3^, which was comparable to that of the HFD group (14,570.7 ± 927.0 mm^3^). In contrast, the L-SLN group reduced the fat volume to 9985.5 ± 1744.1 mm^3^, while the D-SLN group achieved an even more significant reduction, decreasing it to 6818.7 ± 258.3 mm^3^. In terms of dose-dependent effects, D-SLN demonstrated remarkable efficacy.

A clear concentration–response relationship was observed for D-SLN and L-SLN (Figure 3C). At doses of 33.3 mg/kg, 66.6 mg/kg, and 100 mg/kg, D-SLN achieved 33.13%, 53.20%, and 61.80% reductions in total volumes, respectively (*p* < 0.05 vs. HFD). Specifically, subcutaneous fat volume decreased by 38.01%, 57.78%, and 65.93%, while visceral fat volume was reduced by 24.76%, 45.3%, and 54.70% at doses of 33.3 mg/kg, 66.6 mg/kg, and 100 mg/kg. A similar, yet less pronounced, dose-dependent trend was also evident in the L-SLN treatment group. This dose dependency aligns with the hypothesis that SLNs enhance fucoxanthin’s bioavailability and sustained release, enabling prolonged activation of lipolytic pathways such as AMPK and UCP1 [27].

### 2.4. Metabolic and Hepatic Improvements via Serum Analysis

**Figure 4:**
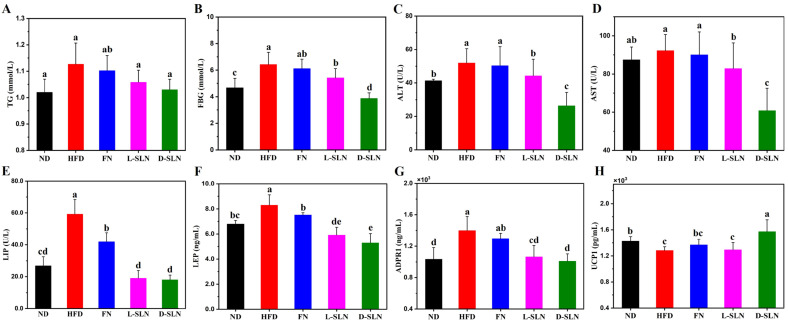
Serum levels of metabolic biomarkers in mice, treated with 6.67mg/kg fucoxanthin-SLNs. (**A**) Triglyceride (TG); (**B**) Fasting blood glucose; (**C**) Alanine aminotransferase (ALT); (**D**) aspartate aminotransferase (AST); (**E**) Lipase (LIP); (**F**) Leptin (LEP); (**G**) Adiponectin receptor 1 (ADPR 1); (**H**) Uncoupling protein-1 (UCP-1). Data are expressed as mean ± SD, n = 5; bars without sharing the same letter are significantly different (*p* < 0.05).

Biochemical and molecular analyses revealed consistent dose-dependent trends, which were closely correlated with the efficacy of various formulations (Figure 4). Among them, the D-SLN group demonstrated the most remarkable improvements in key metabolic parameters. For example, at a dose concentration of 66.67 mg/kg, compared with HFD group, triglyceride (TG) levels in the D-SLN group decreased by 8.58% (*p* < 0.05), fasting blood glucose (FBG) level dropped by 39.61% (*p* < 0.01), and liver injury markers alanine aminotransferase (ALT) and aspartate aminotransferase (AST) dropped by 6.61% (*p* < 0.05) and 34.06% (*p* < 0.01), respectively. Lyophilized SLNs (L-SLNs) exerted moderate effects, whereas unencapsulated fucoxanthin (FN) had a relatively minor impact on these indices.

Adipokines play a pivotal role in regulating energy metabolism, appetite, and immune responses, and their dysregulation is closely associated with obesity and related metabolic disorders [28]. Adipokine profiling in this study demonstrated restored hormonal balance in SLN-treated mice, highlighting the potential therapeutic effects of fucoxanthin-loaded solid lipid nanoparticles. Serum leptin (LEP), a satiety-regulating adipokine often elevated in HFD-induced obesity, was significantly decreased by 28.80% in the L-SLN group (*p* < 0.05) and 36.39% in the D-SLN group (*p* < 0.05). Concurrently, lipase (LIP) levels in D-SLN group declined by 69.48% (*p* < 0.01). The coordinated decrease in both leptin and lipoprotein levels indicated a synchronized regulation of multiple adipokine signaling pathways, indicating a coordinated regulation of multiple adipokine signaling pathways.

Inflammatory and thermogenic pathways were also modulated by SLN formulations. Fresh D-SLN treatment potently activated thermogenesis, as evidenced by a 22.53% upregulation of uncoupling protein-1 expression (UCP-1). Additionally, levels of Adiponectin receptor 1 (ADPR 1), a protein associated with cellular stress and metabolic regulation, exhibited a hierarchical decrease across the HFD and SLN treatment groups, with the most pronounced reduction observed in D-SLN formulations.

### 2.5. MCP1 Protein Expression in Liver

**Figure 5:**
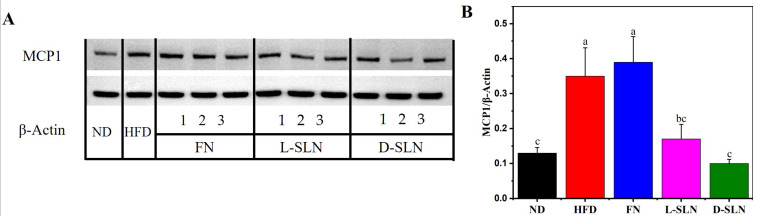
(**A**) Western blot analysis of MCP1 protein in liver. (**B**) Level of MCP1 protein expression relative to β–actin, illustrated with the dose concentration of 66.67 mg/kg. Data are expressed as mean ± SD, n = 5; bars without sharing the same letter are significantly different (*p* < 0.05).

The anti-inflammatory effects of fucoxanthin-loaded solid lipid nanoparticles (FN-SLNs) were evaluated by measuring the MCP1/β-actin ratio in the liver through western blot analysis. In HFD-induced obese mice, the MCP1/β-actin ratio was significantly higher (0.35) compared to the normal diet (ND) group (0.13, *p* < 0.05), reflecting an enhanced inflammatory state in the HFD model. Notably, administration of free fucoxanthin failed to reduce the MCP1/β-actin ratio; instead, a marginal increase to 0.39 was observed in the FN-treated group, indicating limited anti-inflammatory efficacy. Conversely, both lyophilized SLNs (L-SLNs) and dispersed SLNs (D-SLNs) demonstrated significant anti-inflammatory effects. The MCP1/β-actin ratio decreased to 0.17 in the L-SLN group and 0.10 in the D-SLN group, representing a 51.4% and 71.4% reduction relative to the HFD group, respectively (*p* < 0.05 for both comparisons) (Figure 5A,B). These results clearly demonstrate that SLN encapsulation substantially enhances fucoxanthin’s ability to suppress hepatic inflammation, with D-SLN exhibiting superior anti-inflammatory activity compared to free fucoxanthin.

### 2.6. Histopathological Analysis 

**Figure 6:**
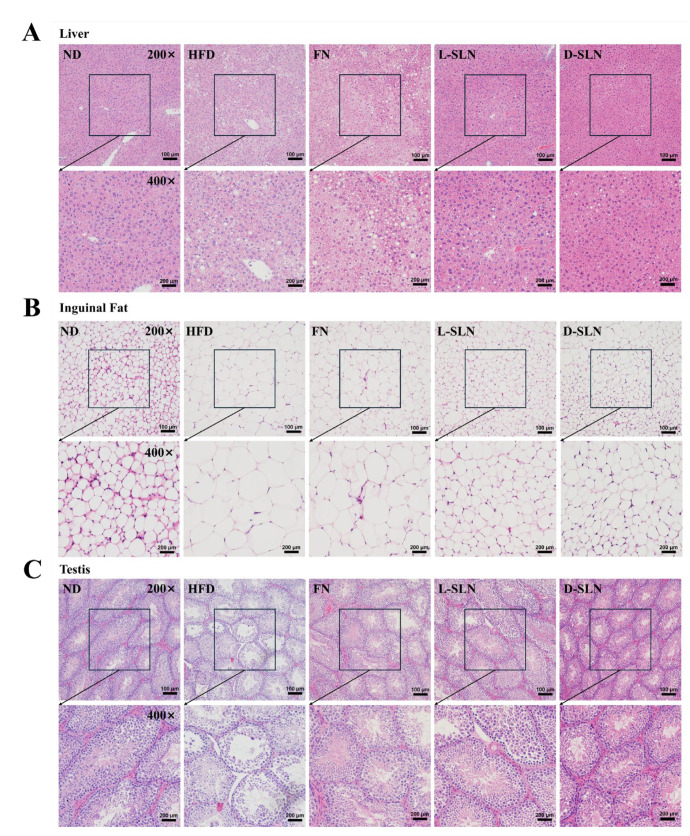
Hematoxylin–eosin (H&E)-stained tissue sections visualized under a light microscope at 200× magnification. (**A**) Liver tissue; (**B**) inguinal adipose tissue; (**C**) testis tissue.

Under the light microscope, H&E-stained liver sections of normal diet (ND) mice displayed healthy and well-organized hepatic architecture (Figure 6A). In contrast, the high-fat diet (HFD) mice presented profound pathological changes. Extensive microvesicular steatosis, along with some macrovesicular steatosis, was evident. Mice treated with free fucoxanthin (FN) showed histological features comparable to those of the HFD group with a large number of lipid droplets accumulated in hepatocytes. Notably, the intervention with solid lipid nanoparticles (SLNs) encapsulating fucoxanthin led to substantial improvements. The degree of steatosis was markedly reduced, with fewer and smaller lipid vacuoles observed in hepatocytes. Among them, mice treated with D-SLN demonstrated superiority over L-SLN.

As illustrated in Figure 6B, adipocytes exhibited a regular arrangement, with small and highly uniform sizes in the ND group. In contrast, significantly enlarged adipocytes were observed in the HFD group. FN-SLNs normalized adipocyte morphology, comparable to the normal-diet control group. Testicular histology in HFD mice showed seminiferous tubule disorganization, germ cell layer loss, and interstitial edema (Figure 6C). FN-SLNs preserved spermatogenic architecture, restored germ cell differentiation, and decreased apoptosis to levels approaching those of ND groups.

## 3. Discussion

Obesity is a prevalent health issue with a high global burden, intricately linked to various metabolic disorders. Fucoxanthin, a bioactive compound sourced from marine algae, has shown promise in anti-obesity research. However, its practical application is hindered by poor solubility and unfavorable pharmacokinetic properties. To overcome these challenges, the development of fucoxanthin-loaded solid lipid nanoparticles (SLNs) as a targeted anti-obesity therapy has been an area of active investigation. This study advanced the development of FN-SLNs as a targeted anti-obesity therapy, building on our prior work of an innovative drug delivery system designed and proved with an in vitro release profile and in vivo pharmacokinetic study [22]. By comparing free fucoxanthin (FN), freshly dispersed SLNs (D-SLNs), and lyophilized SLNs (L-SLNs), we demonstrated that nanoencapsulation significantly enhanced therapeutic efficacy through improved bioavailability, targeted delivery, and modulation of key metabolic–inflammatory pathways.

Oral administration of fucoxanthin formulations elicited hierarchical improvements in obesity-related parameters in a formulation-dependent manner, with D-SLN > L-SLN > FN in reducing serum triglycerides, fasting glucose, and hepatic transaminases (ALT, AST) (Figure 4).

Notably, D-SLN at the lowest dose (33.33 mg/kg) was able to achieve a reduction in body weight and fat mass equivalent to the highest FN dose (100 mg/kg) (Figure 2B and Figure 3C). This finding underscores the critical role of SLNs in overcoming fucoxanthin’s poor solubility and pharmacokinetic limitations. However, when higher doses of D-SLNs were administered, although a reduction in total fat volume was still observed (Figure 3C), there was a plateau in weight loss (Figure 2B). This phenomenon was attributed to an efficacy window governed by biological targets, such as saturation of β3-adrenergic receptors in adipose tissue or capacity limits of metabolic pathways [29].

The significant reduction in body weight and fat mass by FN-SLNs was associated with multi-faceted metabolic improvements. Biochemical analyses revealed that SLNs effectively decreased serum triglycerides, fasting glucose, and liver enzymes, indicating enhanced lipid metabolism, glycemic control, and hepatoprotection (Figure 4). These effects may be mediated by the activation of peroxisome proliferator-activated receptor alpha (PPARα), which promotes fatty acid oxidation, and the suppression of the nuclear factor kappa-light-chain-enhancer of activated B cells (NF-κB) pathway, reducing inflammation and oxidative stress [30]. Additionally, the normalization of adipokine levels, including leptin and adiponectin, suggests restored hormonal balance and improved insulin sensitivity in SLN-treated mice [31,32].

The modulation of thermogenic and inflammatory pathways further contributes to the therapeutic effects of FN-SLNs. The upregulation of uncoupling protein-1 (UCP-1) in adipose tissue indicates enhanced energy expenditure and browning of white adipocytes, which is crucial for combating obesity [33]. Meanwhile, the reduction in pro-inflammatory cytokines of monocyte chemoattractant protein-1 (MCP-1) suggested alleviation of adipose tissue inflammation [34]. These findings are consistent with previous studies demonstrating fucoxanthin’s ability to regulate thermogenesis and inflammation through the activation of β3-adrenergic receptors and the suppression of pro-inflammatory pathways [33].

The histological evaluation of hepatic, adipose, and testicular tissues provides additional insights into the protective effects of FN-SLNs. In the liver, SLNs prevented HFD-induced steatosis and inflammation, likely by promoting lipid metabolism and reducing oxidative stress [35]. In adipose tissue, FN-SLNs inhibited adipocyte hypertrophy and promoted browning, contributing to the reduction in fat mass. Notably, the preservation of testicular architecture and function in SLN-treated mice suggests potential benefits for reproductive health, which warrants further investigation [36]. These histological findings complement the biochemical and molecular data, underscoring the multi-organ protective effects of FN-SLNs.

In contrast to Mi-Bo Kim et al. [16], who reported that low-dose free fucoxanthin failed to mitigate hepatic and adipose inflammation or fibrosis in diet-induced obese mice, our findings showed that the SLN delivery system conferred efficacy even at equivalent low doses. This divergence reflected that encapsulation enhanced fucoxanthin stability, facilitated lymphatic uptake to bypass hepatic clearance, and enabled sustained release in target tissues.

While both SLN formulations outperformed FN, D-SLN exhibited superior efficacy. This disparity can be attributed to several critical factors related to formulation stability and delivery efficiency, consistent with previous research on nanoparticle-based drug delivery [37,38]. The lyophilization process induces aggregation of nanoparticles, increasing the average particle size [39]. This size increase reduces the lymphatic targeting efficiency of L-SLN, as smaller particles are more readily taken up by the lymphatic system and transported directly to adipose tissues, bypassing the hepatic first-pass metabolism [40]. Lyophilization-induced particle aggregation increased the mean hydrodynamic diameter of L-SLN from 237.21 nm (D-SLN) to 261.30 nm, impairing lymphatic uptake efficiency and adipose tissue targeting [41]. These findings highlight the tradeoff between formulation stability (lyophilization for shelf-life) and functional performance (targeting/release kinetics), emphasizing the need for cryoprotectant optimization or surface modification to minimize aggregation and preserve the advantages of fresh SLNs in future studies.

The current study demonstrated the promising potential of FN-SLNs in mitigating high-fat diet induced obesity and related metabolic disorders. Future studies on elucidating the efficacy ceiling (such as target receptor saturation or metabolic pathway limitations) and optimizing the lyophilized formulation would be valuable for translating the therapeutic potential of FN-SLNs into clinical practice.

## 4. Materials and Methods

### 4.1. Materials

Free Fucoxanthin powder (purity ≥ 98%) was purchased from Kari Marine Technology Co., Ltd. (Rizhao, China). Coconut oil and Tween 80 were purchased from Macklin Biochemical Co., Ltd. (Shanghai, China). Glyceryl monostearate was purchased from Aladdin Bio-Chem Technology Co., Ltd. (Shanghai, China). HPLC-grade acetonitrile and methanol were obtained from Thermo Fisher (Darmstadt, Germany). HPLC-grade formic acid was obtained from Roe Scientific Inc. (Powell, OH, USA). Ultrapure water was produced by a Millipore Milli-Q system (Millipore Corp., Billerica, MA, USA). All other reagents or solvents were commercially available and reagent grade.

Six-week-old male C57BL/6J mice used in the study were obtained from Zhuhai BesTest Bio-Tech Co., Ltd. (Zhuhai, China). Reagent kits for veterinary serum analysis, including aspartate aminotransferase (AST), alanine aminotransferase (ALT), triglycerides (TG), and lipase (LIP), were purchased from Shenzhen Mindray Animal Medical Technology Co., Ltd. (Shenzhen, China). ELISA kits for the quantification of inflammatory and metabolic biomarkers, including leptin (LEP), uncoupling protein 1 (UCP1), Adiponectin receptor 1 (ADPR1), and Monocyte Chemoattractant Protein-1 (MCP-1) antibody, were purchased from Nanjing Jiancheng Bioengineering Institute (Nanjing, China).

### 4.2. Methods

#### 4.2.1. Fabrication of FN-SLNs

FN-SLNs were fabricated following the method developed in our previous paper [22], with minor adjustments to enhance fucoxanthin loading. Briefly, lipids phase (95% coconut oil and 5% glyceryl monostearate (GMS), *w*/*w*) were melted in a water bath at 65 °C, followed by the addition of non-ionic surfactants (1.25% Tween 80 and 1.25% soy lecithin, *w*/*w*) and magnetic stirring for 15 min. Crystalline fucoxanthin powder (20% in lipid, *w*/*w*) was incorporated into the lipid phase and stirred for 10 min in the dark until fully dissolved. The lipid mixture was emulsified with Milli-Q water using a high-speed homogenizer (Ultra Turrax T25, IKA^®^ Works, Inc., Wilmington, NC, USA) at 7800 rpm for 5 min, followed by micro fluidization (MP-110 Microfluidics, Westwood, MA, USA) at 100 MPa for 4 cycles to reduce particle size. The emulsion was cooled to 4 °C to induce lipid recrystallization.

#### 4.2.2. Characterization

FN-SLNs’ particle size, polydispersity index (PDI), and zeta potential were measured by dynamic light scattering (DLS) using a NanoBrook Omni laser diffractometer (Brookhaven Instruments, Holtsville, NY, USA) at 25 °C. Samples were diluted 1:199 in ultrapure water and analyzed at pH 7.0. Encapsulation efficiency and loading capacity were determined by HPLC analysis (Agilent 1290 Infinity LC System, Santa Clara, CA, USA) with a C18 column (4.6 mm × 150 mm, 5.0 µm) and UV detection at 450 nm. Lyophilization was performed using a Labconco FreeZone 6L-84 lyophilizer (Labconco Corporation, Kansas City, MO, USA) under 0.001 mbar vacuum at room temperature, with 20% trehalose as a cryoprotectant.

#### 4.2.3. Animal Diets and Experimental Designs

Five-week-old C57BL/6J mice were obtained from Zhuhai BesTest Bio-Tech Co., Ltd. All the mice were housed in standard laboratory conditions with a controlled temperature (23 ± 2 °C) and a 12 h light–dark cycle. They were acclimatized to the laboratory environment for 7 days prior to the start of the experiment to minimize stress-induced effects. All animal treatments were approved by the Institutional Animal Care and Use Committee (refer to: NIH Publications No. 8023, revised 1978) and were carried out according to the Laboratory Animal Management Regulations of the People’s Republic of China (edition 2017), as well as complying with institutional guidelines.

After acclimating for one week, 55 mice were randomized into 11 groups (n = 5/group), with dietary and intervention details as follows:

1. Group 1 (Normal diet, ND): Fed standard chow.
2. Group 2 (HFD + saline): Fed high-fat diet (HFD, D12492, Research Diets, containing 60% kcal from fat [20% saturated, 40% monounsaturated], 20% kcal from protein, and 20% kcal from carbohydrates, supplemented with 0.2% cholesterol) and gavaged with saline (0.2 mL/day).
3. Group 3–5 (free FN powder): Fed HFD and gavaged with fucoxanthin powder at low (33.33 mg/kg), medium (66.67 mg/kg), and high (100 mg/kg) doses.
4. Group 6–8 (lyophilized fucoxanthin-loaded solid lipid nanoparticles: L-SLNs): Fed HFD and gavaged with L-SLNs at low (33.33 mg/kg), medium (66.67 mg/kg), and high (100 mg/kg) doses.
5. Group 9–11 (freshly dispersed fucoxanthin-loaded solid lipid nanoparticles: D-SLNs): Fed HFD and gavaged with D-SLNs at low (33.33 mg/kg), medium (66.67 mg/kg), and high (100 mg/kg) doses.

Subsequently, the anti-obesity effects of fucoxanthin-loaded solid lipid nanoparticles (fucoxanthin SLNs) were evaluated over an 8-week experimental period.

#### 4.2.4. Physiological Analysis

Body weight and food intake were monitored every three days using a digital scale (Sartorius, BS-224S, Göttingen, Germany). To quantify the relative reduction in weight gain of the fucoxanthin-treated group compared to the control group, the weight gain reduction percentage was calculated as follows:

$$Reduced\;\%=\frac{W(control)-W(treated)}{W\;(control\;)}\times 100\%$$

where W(*control*) and W(*treated*) represent the net weight gain of the control and fucoxanthin treated groups, respectively.

#### 4.2.5. Micro-CT Analysis of Adipose Tissue Depots

Mice were anesthetized using isoflurane (4% induction, 1.5–2% maintenance in oxygen at 0.8–1 L/min) and positioned in a Quantum GX2 micro-CT scanner (Revvity, Pleasanton, CA, USA) to generate 3D images of total body fat, subcutaneous, and visceral adipose tissue. Analysis 14.0 software was used to segment fat tissues (density threshold: −190 to −30 HU). Volumes of subcutaneous and visceral fat were quantified by counting 3D voxels, providing both qualitative visualization and quantitative volume data (mm^3^). Mice were recovered in a warm, oxygenated environment post-scan.

#### 4.2.6. Serum Biochemical Parameters Determination

Fasting blood samples were collected via retro-orbital plexus puncture under isoflurane anesthesia after a 12 h fast. Serum was separated by centrifugation at 3000× *g* for 15 min at 4 °C using an Eppendorf 5810R centrifuge (Eppendorf AG, Hamburg, Germany). Serum biochemical parameters, including aspartate aminotransferase (AST), alanine aminotransferase (ALT), triglycerides (TG), and lipase (LIP), were measured using commercial enzymatic colorimetric assay kits following the manufacturer’s protocols. Analysis was performed on a BS-240 VET automatic biochemical analyzer (Mindray Bio-Medical Electronics Co., Ltd., Shenzhen, China) at 37°C. Absorbance was recorded at specific wavelengths—505 nm for TG, 546 nm for HDL and LDL, and 340 nm for AST and ALT—with results calibrated against standard curves to ensure accuracy.

#### 4.2.7. ELISA for Cytokine Quantification

Serum concentrations of monocyte chemoattractant protein-1 (MCP-1), leptin (LEP), and adiponectin (APN) were quantified using commercial ELISA kits specific to each analyte, following manufacturer protocols. Briefly, 96-well plates pre-coated with target-specific capture antibodies were incubated at 4 °C overnight, blocked with 1% bovine serum albumin (BSA) in phosphate-buffered saline (PBS), and loaded with diluted serum samples (MCP-1 1:100, leptin 1:50, APN 1:200) and calibration standards for 2 h at room temperature. After five washes with PBST (PBS + 0.05% Tween-20), biotinylated detection antibodies and streptavidin-horseradish peroxidase (HRP) conjugate were sequentially added, followed by tetramethylbenzidine (TMB) substrate for color development. Reactions were terminated with H_2_SO_4_, and absorbance at 450 nm was measured using a microplate reader (Varioskan LUX, Thermo Fisher Scientific Inc., Waltham, MA, USA). Target protein concentrations were derived from kit-provided standard curves and normalized to total protein levels via bicinchoninic acid (BCA) assay to ensure inter-sample comparability.

#### 4.2.8. Western Blot for Protein Expression Analysis

Liver tissues were homogenized in ice-cold RIPA buffer using a high-speed cryogenic tissue grinder (Servicebio KZ-5F-3D, Wuhan, China). After centrifugation (Eppendorf 5417R, Hamburg, Germany) at 12,000× *g* for 15 min at 4 °C, supernatants were collected as total protein extracts. Protein concentration was quantified by BCA assay. Equal 30 μg protein aliquots were separated by electrophoresis apparatus (Bio-Rad PowerPac HC, Hercules, CA, USA) and transferred to 0.45 μm PVDF membranes (Millipore IPVH00010, Billerica, MA, USA). Membranes were blocked with 5% non-fat milk in TBST at room temperature for 1 h. They were incubated overnight at 4 °C with primary antibodies against MCP1 and β-actin (loading control). Visualization was performed using clarity western ECL substrate, and the images were captured by a fully automated chemiluminescence image analysis system (Tanon 5200, Shanghai, China). Band intensities were analyzed by Gelpro32 software and normalized to β-actin to determine relative MCP1 levels.

#### 4.2.9. Histopathological Evaluation

Liver, adipose, and testicular tissues were fixed in 10% neutral-buffered formalin, routinely embedded in paraffin, and sectioned into 5 μm-thick slices using a cryostat microtome (Leica, Wetzlar, Germany). Standard hematoxylin and eosin (H&E) staining was performed according to established histological protocols, and tissue sections were visualized under a bright-field light microscope (Olympus BX53, Tokyo, Japan) for morphological assessment. Microscopic examination included qualitative analysis of cellular architecture, tissue integrity, and pathological changes in each tissue type, with images captured at ×200 and ×400 magnification for detailed evaluation.

#### 4.2.10. Statistical Analysis

Data were analyzed using GraphPad prism version 10.4.2. Results were expressed as mean ± standard deviation (x ± s). Intergroup comparisons were performed using one-way analysis of variance (One-way ANOVA) followed by Tukey’s post hoc test. A *p*-value < 0.05 was considered statistically significant.

## 5. Conclusions

This study demonstrates that solid lipid nanoparticle encapsulation significantly enhances the therapeutic efficacy of fucoxanthin in mitigating HFD-induced metabolic dysfunction. Among the SLN formulations, D-SLN outperforms L-SLN due to its superior particle size, lymphatic targeting efficiency, and rapid drug release profile. The FN-SLN formulations, particularly D-SLN, effectively reduced body weight, improved lipid and glucose metabolism, alleviated inflammation, and enhanced thermogenesis in obese mice. The histological evaluation further confirmed the multi-organ protective effects of FN-SLNs. These findings highlight the importance of formulation design, including processing methods, in optimizing the delivery and efficacy of fucoxanthin, providing a strong rationale for the development of FN-SLN-based therapies for obesity and related metabolic disorders. Future research should focus on elucidating the underlying mechanisms, optimizing the formulation, and conducting preclinical and clinical studies to evaluate the safety and efficacy of FN-SLNs for potential clinical applications.

## Figures and Tables

**Table 1:** Comparative physicochemical properties of L-SLN powder and D-SLN.

SLN	Size (nm)	Zeta Potential (mV)	PDI	(EE%)
lyophilized SLN powder	261.30 ± 3.14	−30.60 ± 0.51	0.24 ± 0.01	96.91 ± 2.06
fresh SLN dispersion	237.21 ± 1.75	−32.71 ± 0.22	0.17 ± 0.04	98.17 ± 1.21

Data expressed as mean ± SD (n = 3).

## Data Availability

The raw data supporting the conclusion of this article will be made available by the authors on request.

## Abbreviations

The following abbreviations are used in this manuscript:

SLN	Solid Lipid Nanoparticle
HFD	High-Fat Diet
ND	Normal Diet
L-SLN	lyophilized SLN
D-SLN	dispersed SLN
MCP-1	Monocyte Chemoattractant Protein 1
UCP-1	uncoupling protein-1
PPARγ	peroxisome proliferator-activated receptor gamma
C/EBPs	CCAAT/enhancer-binding proteins
SREBP-1c	sterol regulatory element-binding protein 1c
EE%	Encapsulation Efficiency
AMPK	Adenosine Monophosphate-Activated Protein Kinase
TG	Triglyceride
FBG	fasting blood glucose
ALT	alanine aminotransferase
AST	aspartate aminotransferase
LEP	leptin
LIP	Lipase
ADPR 1	Adiponectin receptor 1
H&E	Hematoxylin–eosin
NF-κB	Nuclear Factor-κB
GMS	glyceryl monostearate
HPLC	High-Performance Liquid Chromatography
